# The Recent Progress and Applications of Digital Technologies in Healthcare: A Review

**DOI:** 10.1155/2020/8830200

**Published:** 2020-12-03

**Authors:** Maksut Senbekov, Timur Saliev, Zhanar Bukeyeva, Aigul Almabayeva, Marina Zhanaliyeva, Nazym Aitenova, Yerzhan Toishibekov, Ildar Fakhradiyev

**Affiliations:** ^1^S.D. Asfendiyarov Kazakh National Medical University, Almaty, Kazakhstan; ^2^NJSC “Astana Medical University”, Nur-Sultan, Kazakhstan; ^3^Institute of Experimental Biology, Almaty, Kazakhstan

## Abstract

**Background:**

The implementation of medical digital technologies can provide better accessibility and flexibility of healthcare for the public. It encompasses the availability of open information on the health, treatment, complications, and recent progress on biomedical research. At present, even in low-income countries, diagnostic and medical services are becoming more accessible and available. However, many issues related to digital health technologies remain unmet, including the reliability, safety, testing, and ethical aspects.

**Purpose:**

The aim of the review is to discuss and analyze the recent progress on the application of big data, artificial intelligence, telemedicine, block-chain platforms, smart devices in healthcare, and medical education. *Basic Design*. The publication search was carried out using Google Scholar, PubMed, Web of Sciences, Medline, Wiley Online Library, and CrossRef databases. The review highlights the applications of artificial intelligence, “big data,” telemedicine and block-chain technologies, and smart devices (internet of things) for solving the real problems in healthcare and medical education. *Major Findings*. We identified 252 papers related to the digital health area. However, the number of papers discussed in the review was limited to 152 due to the exclusion criteria. The literature search demonstrated that digital health technologies became highly sought due to recent pandemics, including COVID-19. The disastrous dissemination of COVID-19 through all continents triggered the need for fast and effective solutions to localize, manage, and treat the viral infection. In this regard, the use of telemedicine and other e-health technologies might help to lessen the pressure on healthcare systems. *Summary*. Digital platforms can help optimize diagnosis, consulting, and treatment of patients. However, due to the lack of official regulations and recommendations, the stakeholders, including private and governmental organizations, are facing the problem with adequate validation and approbation of novel digital health technologies. In this regard, proper scientific research is required before a digital product is deployed for the healthcare sector.

## 1. Introduction

The classic healthcare model is predominantly based on providing medical services through the systems of hospitals and outpatient clinics. The quality of the health service depends on many factors such as the qualification of medical personnel, hospital facilities, and the availability of up-to-date equipment. The model may vary from country to country. However, the core principles remain the same. First of all, it concerns the “patient-oriented” approach and supporting infrastructure that provide optimal access to the healthcare service. In recent decades, such a classic platform has been experiencing new challenges due to the rapid growth of technologies and the demand of the population in high-quality medical service. Moreover, novel digital technologies offer the possibility of explosive expansion of the potential of various diagnostic and therapeutic tools and systems [1].

In fact, the implementation of medical digital technologies can provide better accessibility and flexibility of healthcare for the general public. It includes the availability of open information on the health, treatment, complications, and biomedical research in the Internet. On the other hand, the diagnostic and medical consulting services are becoming more accessible and available even in low-income countries [2, 3]. Telemedicine provides an opportunity for people from rural and remote regions to get high-quality consulting and advice, while online pharmacy platforms allow obtaining the necessary drugs without unnecessary commuting [4, 5].

Another promising and rapidly growing area is the application of artificial intelligence (AI) in biomedicine, healthcare, and medical education. AI can help to significantly improve the performance and ability of diagnostic platforms. Moreover, it could contribute to the optimization of treatment processes thus leading to an increase in therapeutic efficiency, patient satisfaction, and lower costs [6]. AI can also facilitate conducting biomedical experiments and clinical trials [7]. In addition, AI will be indispensable in the areas requiring automation and intense physical labour. However, despite the recent progress, at present, AI cannot fully replace humans in the field of healthcare and biomedical research.

**Figure 1:**
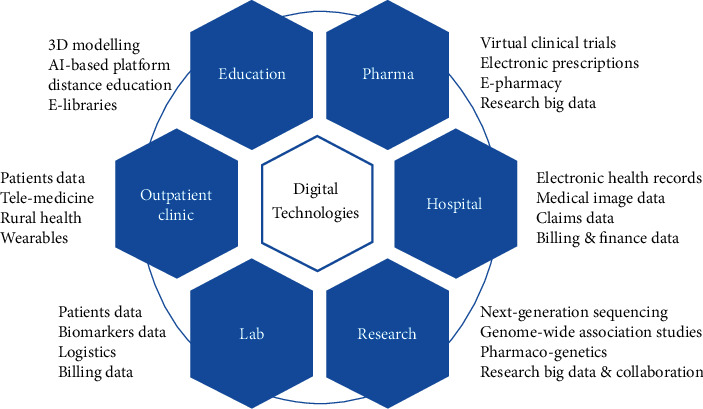
Scheme of main applications of digital technologies in healthcare.

This review discusses the recent trends and achievements in the field of digital medical technologies. It covers the applications of artificial intelligence, “big data,” telemedicine, and block-chain technologies for solving the real problems in healthcare and medical education (Figure 1).

This paper reviews and discusses recent trends and achievements in the field of digital health by reviewing publications focused on the application of artificial intelligence, “big data,” telemedicine, and block-chain technologies, as well as smart devices (“internet of things”) for solving the real problems in healthcare and medical education (Figure 1). The opportunities and main challenges in these areas are examined and intensively discussed. So far, we have identified more than 252 publications related to the digital health area. Nonetheless, the actual number of papers discussed in the article is limited to 152 due to the exclusions criteria. In fact, the literature search shows that digital health technologies became highly sought due to recent pandemics, including COVID-19. The fast spread of COVID-19 through all countries has stimulated the necessity to find solutions to prevent, localize, and treat the life-threatening viral infections. It encompasses the application of AI-based platforms to help with the identification of epidemiologic risks. It opens an avenue for the effective prediction, prevention, and detection of future global health risks, including COVID-19.

Another promising direction in medical research is virtual clinical trials, which have several advantages over the traditional model. Unlike standard clinical trials that require frequent visits, the virtual clinical trials model is based on the monitoring of the patient at home. It provides an opportunity for people from rural regions or those with mobility problems to take part in the studies. It becomes especially relevant during pandemics and cataclysms of natural or man-made origin.

In addition, the review discusses the recent progress on 3D printing. It encompasses the discussion about the application of 3D printing technology for manufacturing models of organs, permanent implants, testing medical devices, personalized 3D drug printing, and medical education.

The review is structured as follows. The first subchapter discusses the recent trends and progress on the application of Artificial Intelligence in public health and medical education. This part of the paper encompasses examples of clinical applications of AI in healthcare, including the use of smart devices for heart monitoring. The second subchapter is focused on big data and E-health applications in medicine and education. The part of the review provides an analysis of the fast-growing field of block-chain technology in healthcare. The next subchapters are organized in the following order: smart-devices, virtual clinical trials, telemedicine, and 3D printing. The fourth chapter “Discussion” highlights and discusses the results, achievements, problems, recommendations, and study limitations. The last part of the review provides a conclusion.

## 2. Methods

### 2.1. Search Strategy

The publication search was carried out using Google Scholar, PubMed, Web of Sciences, Medline, Wiley Online Library, and CrossRef databases (peer-reviewed publications in English).

### 2.2. Inclusion and Exclusion Criteria

For review and analysis, we included articles related to the digital health area that have been published from 2000 to 2020 in the English language. The keywords used for the search were the following: big data, artificial intelligence, telemedicine, internet of things, block-chain, wearables, smart devices, medical education, virtual clinical trials, and 3D printing.

The conducted literature research was solely restricted to available databases (electronic and printed) and did not consider any human subjects related data (e.g., clinical, biological).

**Figure 2:**
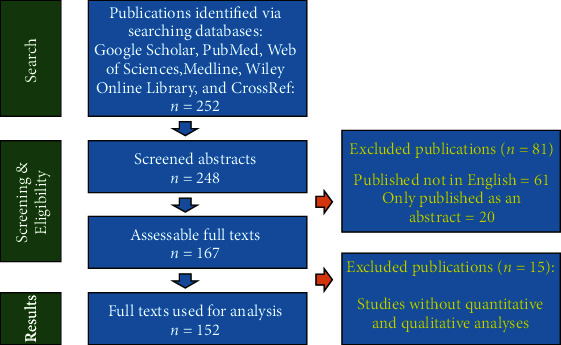
Flow chart summarizing the search process and results.

We identified 252 papers. However, the number of papers discussed in the review was limited to 152 due to the exclusions criteria (Figure 2). In the following chapters, we provide a review and an analysis of the current situation and of the different perspectives, with special emphasis on solving problems related to the vital and main aspects of digital health technologies, including information privacy, data handling, accessibility, availability, and cost-efficiency (particularly for low-income countries) which are up to date unsolved.

## 3. Results

### 3.1. Artificial Intelligence in Public Health and Medical Education

The term “artificial intelligence” (AI) has been associated with mimicking human intelligence or cognitive functions by computers [8]. There is an almost endless range of possible AI applications in the various fields of industries. One of the promising and rapidly growing modern trends is the use of the capabilities of AI and Machine Learning (ML) in healthcare, including the diagnosis and treatment of a number of diseases [9, 10]. The main areas of the applications of AI in medicine are health monitoring, managing patients data, drug development, surgery, remote consultation, medical statistics, personalized treatment, and imaging [11] (Table 1).

Up to date, one of the main areas for AI application in healthcare is radiology. In fact, it is possible to significantly increase the efficiency of medical operation capacities through self-training of machines for reading images (MRI, CT, and ultrasound) and their quick analysis in order to make an accurate diagnosis and treatment planning [20, 21]. AI can help radiologists with image acquisition and reconstruction [22]. For instance, recently, GE Healthcare and Canon Medical Systems started an ML-based programme of image reconstruction for CT scanners. The project is aimed at decreasing the radiation dose without affecting the quality of images [22]. Another example concerns the products of the company Subtle Medical (USA) for the optimization of PET and MR imaging processes. Their systems SubtlePET™ and SubtleMR™ are the first AI solutions approved by the FDA for the enhancement of medical imaging. These systems are based on learning algorithms that are able to communicate and integrate with PET and MRI machines to optimize image recognition and analysis without interruption with the existing workflow.

Apart from radiology, AI can be extremely useful in primary care as well. It encompasses the application of AI platforms for improving diagnostics, practice management, clinical decision making, and education of primary care professionals [23]. Pritesh Mistry (Royal College of General Practitioners, UK) classified the AI use for primary care into two categories: (1) clinical decision making and care management, including symptom assessment, automation of clinical coding, image recognition for dermatological conditions, triaging, and self-management; and (2) proactive detection (analyzing patient records to predict patients with undiagnosed conditions) [24]. It was shown that AI can be used for predictive modeling in forecasting hospital mortality, unplanned readmission, unnecessary long hospital stay, and treatment cost-effectiveness [25]. The driving forces for the implementation of AI in primary care are private medical companies. In the recent decade, some companies such as Babylon Health and Ada developed special platforms for providing service for the patients, including remote consultations via mobile applications [25]. Other companies offer various types of wearable devices integrated with AI. Such devices can be very helpful for primary care physicians to collect and analyze health data thus saving valuable time and resources.

The AI-based platforms demonstrated an ability to improve and optimize the work of cardiologists. It was shown that novel mobile sensors can help cardiologists to monitor, interpret, analyze, and respond to the request based on the biomedical data collected remotely and automatically from the patient [26–31]. At present, diagnostics of heart failure have been mainly based on the analysis of patient's history, their physical examination, and both laboratory and imaging data (ultrasound, CT, and MRI). In this regard, AI-based systems can help to improve diagnosis through leveraging data found from each of these areas, including electrocardiography, echocardiography, electronic health record data, and other sources [27]. There is a range of studies on the use of AI and machine-learning platforms in cardiology. For example, Attia et al. studied the possibility of the application of AI to the electrocardiogram (ECG), a routine method of measuring the heart's electrical activity, for the diagnosis of Asymptomatic left ventricular dysfunction (ALVD) [32]. The authors trained a convolutional neural network to identify patients with ventricular dysfunction, defined as ejection fraction ≤ 35%, using the ECG data alone. The network model yielded values for the area under the curve, sensitivity, specificity, and accuracy of 0.93, 86.3%, 85.7%, and 85.7%, respectively. In patients without ventricular dysfunction, those with a positive AI screen were at 4 times the risk (hazard ratio, 4.1; 95% confidence interval, 3.3 to 5.0) of developing future ventricular dysfunction compared with those with a negative screen. The authors concluded that the application of AI to the ECG—a ubiquitous, low-cost test—permits the ECG to serve as a powerful screening tool in asymptomatic individuals to identify ALVD [32]. A list of examples of the applications of smart devices for heart monitoring is provided in Table 2.

In the light of recent challenges related to COVID-19 pandemics, AI-based platforms can help to identify and reduce epidemiologic risks [42, 43]. Such an approach provides an opportunity for the effective prediction, prevention, and detection of future global health risks, including infections [44–47]. In fact, the search in the Web of Science database (using keywords: “artificial intelligence” and “COVID-19”) resulted in more than 200 publications. It indicates the interest and urgent need in the application of AI and machine learning to solve the problems related to the spread of this life-threatening infection. One of main the problem associated with COVID-19 is the prioritization of patients with COVID-19. It is a complex issue due to various inclusion and exclusion criteria for patient sorting. In a recent study, Albahri et al. proposed a novel multibiological laboratory examination framework for prioritizing patients with COVID-19 on the basis of integrated multicriteria decision-analysis (MCDA) methods [44]. The experiment was conducted on the basis of three phases. In the first phase, patient datasets containing eight biological laboratory examination criteria for six patients with COVID-19 were derived and discussed. The outcome of this phase was used to propose a decision matrix on the basis of the intersection between “biological laboratory examination criteria” and “COVID-19 patients list.” In the second phase, the analytic hierarchy process (AHP) method was utilized to set the subjective weights for the biological laboratory examination criteria by respiratory experts. In the last phase, the VIekriterijumsko KOmpromisno Rangiranje (VIKOR) method was adopted to prioritize patients in the context of individual and group decision making (GDM). Results showed that the integration of the AHP–VIKOR method based on individual and GDM contexts was effective for solving prioritization problems for patients with COVID-19, and the prioritization results of patients with COVID-19 showed no variation in the internal and external VIKOR GDM contexts [44]. The proposed multibiological laboratory examination framework was able to differentiate between the mild and serious or critical conditions of patients with COVID-19 by prioritizing them based on integrated AHP–VIKOR methods.

Another promising field for AI application is medical education. In fact, the scope of medical knowledge and data is growing very rapidly. Moreover, there is a lack of time for health professionals for continuous education and self-improvement. Medical education mostly relies on patient care, medical knowledge, communication skills, practice-based learning, professionalism, and systems-based practice [48, 49]. The significant piece of any medical education is predominantly based on the memorization and is time-consuming [49]. In this case, some tasks can be addressed and delegated to AI-based platforms. Moreover, AI-based training in medical education can complement and enrich the current curriculum. So the students will have a chance to learn how to use AI tools for understanding the main principles and solving real clinical problems [49].

AI-based education can be successfully employed in situations where classic approaches do not work efficiently anymore. For instance, in some countries, the anatomy studies have limitations due to cultural or religious restrictions. In this regard, AI technologies (such as augmented vision) might be used to provide full information on human anatomy and physiology. It can also facilitate the interaction between students and lectors around the globe for the exchange of ideas and optimization of education processes.

Several authors claim that AI will have a significant impact on medical education in the close future. Recently, Chan and Zary stressed out that little emphasis was placed on the revision of medical students' curriculum and assessment of learning progress due to the lack of digitalization and sensitive nature of examinations [50]. The authors proposed to introduce AI into the medical school's curriculum to better understand AI algorithms and benefits. Indeed, AI-based educational platforms can facilitate solving multidimensional and multidisciplinary problems, establishing relationships between variables, and provide greater classification accuracy [50].

Another study demonstrated the effectiveness of an AI-based simulator with a statistically significant, 22% improvement of the posttraining diagnostic accuracy, as compared to a multimedia-based, expert-led training with a nonstatistically significant improvement of only 8%.

The main barriers to the widespread application of AI for medical education and training are limited digitalization and financial constraints. Such a situation is not only common for undeveloped countries but also for Western institutions as well [50]. The problem can be explained by the prevalence of classic approaches in medical education and the resistance of educators to the changes and novel methodologies. Another factor delaying the implementation of AI-based educational platforms is the weak involvement of private companies in such programmes.

Another big area for AI application in healthcare is cybersecurity due to the massive amount of personal data accumulated during recent years. Despite the many benefits of digital technology, they can also lead to data vulnerabilities, so implementing an effective AI security solution is crucial [51].

### 3.2. Big Data and E-Health

According to Gartner Glossary: “Big Data is high-volume, high-velocity and/or high-variety information assets that demand cost-effective, innovative forms of information processing that enable enhanced insight, decision making, and process automation.” Big data refers to the large, diverse sets of information that grow at ever-increasing rates. It encompasses the volume of information, the velocity or speed at which it is created and collected, and the variety or scope of the data points being covered.

In recent decades, big data has been increasingly used to improve and optimize the management, analysis, and forecasting in healthcare [52, 53]. For example, the transition to Electronic health records (EHR) can help to store, sort, and speed up the processing of patients' data. Moreover, the application of big data systems in the management of medical practice has the potential to improve the quality, the efficiency of the service, lower the cost of care, and the number of medical errors. In fact, the recent widespread use of EHR has led to the explosive growth of big data in healthcare. Apart from the pure clinical field, big data applications in healthcare also cover data on health care providers (pharmaceuticals and logistics), genetics, and other information [54].

There is a steady global trend for the use of big data as a platform that provides means to support, optimize, accelerate, and facilitate biomedical research. It also provides unlimited opportunities for finding new solutions in biology, medicine, genetics, epidemiology, and pharmacology [53, 55]. Big data can help with sorting and analysis of the rapidly increasing volume of medical information. For instance, clinical data from one person can generate 0.4 terabytes of information (per lifetime), genomics data around 6 terabytes, and additional data of 1,100 terabytes [56]. It was estimated that by 2020, the volume of medical data will double every 73 days [56]. In addition, the platforms based on big data can solve the issues related to velocity (analysis of streaming data) [57], variety (different forms of data) [54], and veracity (uncertain of data) of the public health data.

Big data can be employed for the analysis of data from the next-generation sequencing (“next-generation sequencing,” NGS) and the study of wide genome association (“genome-wide association studies,” GWAS) for decoding human genetics. The new era of “-omics” (based on big data platform) opens an opportunity to explore and understand the whole “genome,” thus generating a large amount of information with a greater depth of understanding [54].

In fact, healthcare requires a strong integration of biomedical data from various sources such as electronic health/medical records, genetics, information for pharmacies, and insurance records in order to optimize diagnostics and treatment via a personalized approach. The analysis of big data from medical systems can be also useful for the development of novel strategies for healthcare and create the prerequisites for fundamental changes in the field of personalized medicine in the near future [54].

Up to date, in many countries, the concept of e-health has already been developed and officially approved. E-health platforms allow to receive (automatically and timely) and analyze medical information, thus providing safe, fair, high-quality, and sustainable healthcare services focused on the patients' needs. Currently, the world-leading health organizations are actively taking measures to introduce and standardize the Electronic Health Passport to strengthen primary health care. It facilitates the improvement and the optimization of the quality of medical care along with reducing the amount of paperwork for a medical professional. Standardization is necessary to ensure the functional interaction between the electronic health passport, electronic medical records, and other e-health systems, taking into account the requirements of standard ISO 13940 : 2015. Electronic documentation systems can accumulate information about the patient throughout his/her life with personal access of the citizen (patient) to the following data: information about clinical observations, medical history, information about treatment, vaccination, prescribed drugs, allergic reactions, symptoms, health status, and results from diagnostic studies. In addition, the introduction of e-health systems will allow doctors to write out and send electronic prescriptions directly to the pharmacy network, which will also significantly reduce the problem of patient queues in clinics and paperwork.

In fact, each patient generates myriads of data, such as information about a diagnosis, treatment, drugs, medical supplies, images, lab results, and financial documentation. In this regard, the big data platform could considerably improve the functionality, security, analyzing, and performance of e-health systems. At present, some companies have proposed technologies based on big data solutions such as STORM and Hadoop [58].

Nevertheless, it should be noted that the current poorness of IT infrastructure and equipment of medical organizations does not allow for the full-scale implementation of medical information systems, especially in undeveloped countries. In fact, in most regions in poor countries, with the exception of the central regions, the workplaces of medical personnel are not equipped with personal computers, and there is no structured cabling system and server equipment.

Taking into account the rapid growth and importance of big data for healthcare, medical education has to incorporate the training on big data usage in the curriculum. For example, the application of big data can help to improve the clinical experience of medical trainees and interns. Moreover, advanced methods based on big data might help to solve some fundamental issues related to medical education [59]. For instance, machine learning may be applied to big data analytics to optimize the number of procedures a trainee (resident) has to conduct in order to achieve an optimal completion rate [59].

### 3.3. Block-Chain Technology for Healthcare and Education

Block-chain technology is based on a peer-to-peer platform that provides an opportunity to securely store the information on thousands of servers. This information can be simultaneously used and shared within a decentralized and open network. Such an approach makes it difficult for the user to control or change it. Thus, block-chain technology with unique characteristics, such as decentralization, transparency, and anonymity, has been increasingly used in healthcare. According to IBM, 70% of healthcare leaders predict that the block-chain's greatest influence in healthcare will be to improve clinical trial management, regulatory compliance, and a decentralized structure for sharing electronic medical records. The global market for healthcare block-chain technology is expected to exceed $500 million by 2022 [60, 61].

Currently, medical institutions are experiencing an increased demand for real data from industrial and research organizations. At the same time, unauthorized exchanges, widely publicized hacks, and robbery of confidential data constantly undermine public trust in healthcare facilities. The third problem is abuses in the healthcare ecosystem that share the same trust (e.g., problems with fake drugs, procedures, skills, and patients) [60]. Taken together, this is a situation that requires rethinking and consideration of alternative approaches. Thanks to some of its key attributes, such as decentralization, distribution, and data integrity, as well as without any necessary third party, block-chain technology has many attractive properties that can be used to improve and obtain a higher level of interaction, information exchange, access control, and the origin and integrity of the data among the mentioned stakeholders, thereby moving towards a new infrastructure for building and maintaining trust [62].

Ensuring the safety and protection of important medical data is currently the main application for the block-chain. Data security is a major public health concern. Between 2009 and 2017, more than 176 million patient records were affected by data leaks (including medical and genomic information). The ability of the block-chain to provide reliable and decentralized storage of all patient data makes this technology optimal for security. In addition, the block-chain allows hiding the patient's identity with the help of complex and secure codes that can protect medical data. The decentralized nature of the technology also allows patients, doctors, and healthcare providers to share the same information quickly and safely [62].

Another promising area for block-chain application is medical education [63]. Indeed, block-chain might help medical educators to optimize and trace the impact on the students' monitoring of the most popular learning modules. Moreover, the block-chain platform will provide an opportunity to compare the methodologies and even persons in different institutions across the globe (competency-based medical education). Another distinctive feature of block-chain technology, decentralization, can help secure keeping the information on medical and education licensing and certifications.

To summarize, block-chain based educational platforms can help to solve problems related to management and education processes [64]. For instance, the university's block-chain system might securely keep the information about academic staff, teaching content, examination outcomes, staff and student performance, and degrees conferred that encompass the integrity and totality of the learning processes. Moreover, block-chain provides a possibility to form and optimize educational infrastructure possessing an information on the origin of the document, date, and authors. It would improve the educator-student interaction and speed up the feedback thus improving the whole educational system. It is also important for the exclusion of the possibility of corruption and can help to enhance management efficiency.

### 3.4. Smart Devices

The practice of using the first generations of smart devices demonstrated that they can play an important role in monitoring the vital functions of the body and diagnostics [65, 66]. Novel technologies, such as radio frequency identification (RFID) readers and Near Field Communication (NFC) devices, can be utilized not only for collecting health information but also as communication platforms in medicine [54]. Such devices can create a continuous data flow, while monitoring the state of health that makes these devices the main source of massive data sets (big data) [67]. Intelligent platforms can connect various devices (“internet of things”) to provide reliable, efficient, and personalized medical care. Using smart and wearable devices, physicians can remotely monitor various health parameters. Therefore, the patients may not need to be hospitalized or visiting a doctor that results in a considerable decrease in healthcare costs.

The recent explosive interest in e-health is also related to the widespread use of mobile telephones and special mobile apps focused on health. Nowadays, smartphones can track various health parameters, especially in combination with wearable devices. In addition to the fact that smartphones can receive primary health data, they can serve as a platform integrating additional sensors [68]. For instance, company Alivecor (USA) developed a range of smart devices that allow recording ECG (KardiaMobile, KardiaPro, and KardiaBand). Such devices can potentially replace traditional bulky ECG monitoring machines, and they can be purchased and owned by the patients [68]. Moreover, the constant monitoring of health parameters such as ECG will provide a possibility of long-term monitoring of heart functions that could be used for qualitative analysis and outcome prediction. Smart devices such as smart watches may help to determine the presence of tachyarrhythmias or bradyarrhythmias [68]. In addition, the information obtained from wearable smart devices can be analyzed at each appointment to predict the recurrence of a supraventricular tachycardia [68]. Recently, the big IT companies offered a range of wearable devices for heart monitoring such as the Apple Watch, Series 4, which is capable of recording and detecting atrial fibrillation, falls, bradycardia, and tachycardia. These smart platforms enable the reduction and prevention of life-threatening conditions such as stroke and infarct.

Wearable devices (“internet of things”) for fitness or health tracking, biosensors, clinical devices for monitoring vital signs, and other types of devices generate a large amount of health-related data. Integration of this data with other existing medical data can help in monitoring health, modeling the spread of pathology, and finding ways to contain the outbreak of a particular disease. In light of recent global challenges, such as the COVID-19 pandemic, the use of smart devices will play an increasingly important role in remote health monitoring [54, 69].

Despite the similarity of the use of smart devices used for fitness and health monitoring, there is a principle difference in the fabrication, methodology, and ethical aspects [70]. First of all, wearable devices for fitness are predominantly designed for personal use (tracking heart functions, etc.). This information can be used by the users to correct and optimize their physical activity [71, 72]. At the same time, medical smart devices are devised to help the physicians to monitor the vital signs and data of the patient. This information can directly affect the speed and quality of diagnostics and treatment outcomes. Besides that, the security and privacy of health data play a crucial role in the application of medical wearable devices (ethical issues) in the clinics [73, 74].

Despite the definite advantages of wearable devices and mobile phones for healthcare applications, there are some concerns raised regarding possible negative biological effects induced by electromagnetic fields generated by such devices [75]. First of all, it encompasses the potential genotoxic [76, 77] and carcinogenic [78, 79] effects of radiofrequency electromagnetic fields (EMF) in the diapason associated with mobile telecommunication systems. However, at the same time, there is a range of reports on the potential therapeutic effect of EMF for different organs and systems, particularly for the brain [80, 81].

Apart from health monitoring, smart devices have been also studied and intensively used for medical education during the recent decade. Snashall and Hindocha pointed out that medical and smart device apps facilitate the concept of situational learning [82]. For instance, it was shown that smartphone facilitated the teaching of anatomy via the use of visual aids, and it led to the enhancement of the learning experience [83]. The study conducted by Bansal et al. revealed that smartphones have been widely used in the medical education system, but their use is still limited to sharing timetables, assignments, and staying connected with teachers and students [84]. The main advantages of smart devices are availability, portability, and easy access to the information making them a strong alternative to traditional forms of education such as lectures and books.

### 3.5. Virtual Clinical Trials

Another new and promising area in medical research is virtual clinical trials [85]. These include technologies used for remote patient health information retrieval, including tablets, smartphone apps, or wearable sensors. Often these platforms have been described as virtual clinical trials, decentralized trials, distance trials, patient-specific trials, or hybrid trials [85]. The process involves recruiting patients, obtaining their consent, and collecting data. A virtual clinical trial is a system when physical sites and direct interaction with patients are not required any more [86, 87].

In fact, virtual clinical trials have several advantages over the traditional model. The latter encompasses several study sites and requires several visits to the patient in order to conduct the study protocol. Unlike field trials that require frequent visits, distance clinical trials are based on the patient being at home, so those with mobility problems, such as older people or patients living in rural areas, can also take part in a test [88]. This becomes especially relevant during pandemics and cataclysms of natural or man-made origin.

Although virtual trials still require support staff to be located on the research site and invested in data collection and analysis platforms, they are potentially significantly more cost-effective than conventional clinical trials [85]. Another advantage of virtual clinical trials is their ability to retain patients involved in the study (typically about 40% of Phase III patients exit the study).

Virtual clinical trials can eliminate the need for frequent field trips and automate data collection, increasing patient engagement and retention. Virtual clinical trials also provide an opportunity to reduce risk in the drug development process. Researchers participating in the trials can access data from remote monitoring devices (smart devices) in a real-time regime, which increases the efficiency of data analysis. Thus, remote monitoring capabilities can facilitate an adaptive approach to clinical trials, allowing you to improve the design of trials based on accumulated data. Decisions to discontinue drug development can also be made faster, which increases patient safety and reduces the cost of failed studies, which, unfortunately, have become quite frequent in the process of drug discovery [89].

### 3.6. Telemedicine

**Figure 3:**
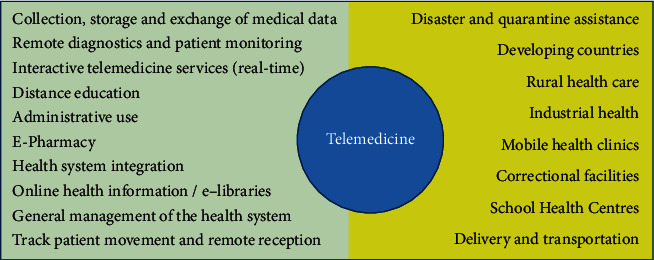
The applications of telemedicine in healthcare.

Telemedicine allows healthcare providers to evaluate, diagnose, and treat patients in remote locations using telecommunication technologies [90, 91]. Advantages of telemedicine include the ability to collect, store, and exchange medical data [92]. Moreover, telemedicine allows remote monitoring of patients, distance education, improving administration and management of healthcare, integration of health data systems, and patient movement tracking [93, 94]. In fact, there are many options for using telemedicine in various sectors (Figure 3).

#### 3.6.1. Disaster and Quarantine Management

Access to medical service after a natural disaster is critical to public health. In this regard, telemedicine allows healthcare workers to quickly analyze and sort the victims. It also provides an opportunity for physicians to remotely contact patients or persons who are in quarantine, thus, avoiding direct contact with the infected patient. Ideally, patients can wear smart devices for the direct transmission of data on the vital functions of the body, including temperature and heart rate. This feature becomes highly requested due to the recent COVID-19 pandemic over the world.

#### 3.6.2. Rural Healthcare

One of the biggest issues in rural healthcare is ensuring access to health services for people from remote regions and countryside [95, 96]. In this case, telemedicine can help to resolve these problems by providing access to high-quality medical services regardless of the patient's location. It can be done by using real-time video conferencing or special web services. This combination of television and medical technology in combination with special wearable devices allows the doctors to contact the patient and provide advice [97]. The physician can examine the patient, check key vital signs and medical history, evaluate, diagnose, and then determine treatment and prescribe the drugs. Such an approach provides an opportunity for quick communication and feedback from the patient. Moreover, it can significantly reduce the need for unnecessary and expensive commuting, which is especially important for regions with severe climatic conditions [98].

#### 3.6.3. Developing Countries

Despite the recent progress in healthcare, the population of many developing countries does not have full access to high-quality medical service yet. In this regard, telemedicine provides an opportunity to introduce novel breakthrough technologies in healthcare through the relatively inexpensive and available healthcare system. Instead of building and maintaining a large number of modern facilities, telemedicine allows basic clinics to consult and share the experience of medical specialists located anywhere in the world. This significantly changes the strategy of providing medical care to a developing country. The platforms that used telemedicine were actively studied and implemented in the African region during the recent decade [99–101]. For example, a recent study employed a low-cost tablet for electroencephalography (EEG) and epilepsy management in the Republic of Guinea (Western Africa) [100]. The study participants underwent EEG twice, and all recordings were scored and analyzed remotely by experts in clinical neurophysiology. The results showed that a tablet-based EEG had a reproducible quality level on repeat testing and was useful for the electroencephalography diagnostics.

#### 3.6.4. Correctional Facilities

In fact, prison inmates demonstrate higher rates of mental illness, chronic medical conditions, tuberculosis, and other infections compared to the general population [102]. Moreover, each country spends a significant amount of money to provide medical services for the prisoners. In this regard, telemedicine-based platforms can help to provide high-quality medical care without low costs and dangers associated with transporting prisoners, as well as with the need for a specialist doctor. Correctional facilities were able to improve access to medical care, significantly reducing the cost of taxpayers for the medical care of prisoners.

However, despite the definite advantages of the application of telemedicine in correctional facilities, only little progress has been done up-to-date. Mateo et al. highlighted the problems that hinder the more widespread implementation of telemedicine in prisons: (1) the lack of information and official guidelines, (2) internet security risks and elevated costs, (3) shortage of resources in the prison health services (that can operate telemedicine system), and (4) the need to maintain two separate electronic clinical history systems, for the prison and regional health services [103].

#### 3.6.5. School Health

In many countries, school-based health clinics or school primary healthcare cabinets are a key point of access to medical care for children [104, 105]. In fact, telemedicine can help to manage various disorders in school-age children, such as asthma, diabetes, and obesity. More importantly, the implementation of telemedicine-based healthcare can assist to decrease the necessity in emergency care and associated costs. In this case, the school nurse plays an important role in providing high-quality medical care, and she/he has to respond professionally to different needs and everyday challenges. Telemedicine allows a school primary care worker to remotely receive an expert's medical advice and assistance. It provides an opportunity for school children to be monitored and treated without missing a school educational programme. Moreover, it can help to minimize costs and time spending for visiting medical specialists.

#### 3.6.6. Industrial Health

All industrial organizations and facilities, such as factories, mines, and oil-gas platforms, are heavily dependent on the health status of the workers. One of the first industries that implemented telemedicine was the oil-gas industry. It has been based mainly on telephone consulting between offshore platforms and onshore medical services, and today it also includes video conferencing and digital medical devices for remote health monitoring [106]. In fact, the industrial companies must respond to the health needs to support thousands of employees situated at different and remote locations. In this regard, telemedicine can help to avoid costly evacuations of the patients and provide quick and optimal diagnostics and appropriate treatment [107].

#### 3.6.7. Medical Education

Apart from real clinical practice, telemedicine has also great prospects for medical education [108, 109]. There is a range of studies on the incorporation of telemedicine into medical education which demonstrated positive and promising results, showing similar or better efficacy compared to traditional educational approach and high enthusiasm reported by the students [110]. The novel educational approach allows students to contact real patients and highly qualified specialists (lecturers) resulting in the improvement of clinical skills [111].

#### 3.6.8. Pharmacies

It has been shown that telemedicine can enhance and optimize the work of community and hospital pharmacies [112–115]. It was demonstrated that telemedicine services provided after-hours by pharmacists in three community hospitals without 24-hour pharmacy services led to the improvement of drug safety since prescribing accuracy was verified [116, 117]. The telemedicine can help to resolve the medication-related problems such as medication and prescription errors. The introduction of digital platforms can reduce problems with drug incompatibility and lower the potential drug adverse effects. Moreover, it was demonstrated that pharmacist interventions were five times more frequent for resolving drug-related problems with a telemedicine service compared with not providing telemedicine services [118].

In fact, telemedicine and telepharmacy are rapidly growing areas of modern healthcare systems around the world. It helps a pharmacist in a remote or rural location to review patient profiles and evaluate prescriptions so that a technician may fill those prescriptions for patients [113]. At present, telepharmacy encompasses drug ordering, patient consultation by phone, medication therapy management, collaborative drug management, central processing and remote order entry, remote supervision of technician dispensing, automated dispensing systems, and medication kiosks with 24/7 pharmacist counselling [113].

#### 3.6.9. Primary Care and General Practice

Telemedicine can greatly improve the work of GPs through better and timely communication with the patients [119, 120]. It can help the GP to take care of and monitor a maximum number of patients in the area, particularly in rural locations. There is evidence that access to care can be improved when patients and families have the opportunity to receive telehealth care at home rather than in-person care in a clinic or hospital [121]. Moreover, the telehealth system enables timely communication between patients or families and care providers that allows self-management and necessary adjustments that may prevent hospitalization [121]. In fact, the application of telemedicine in GP's practice can significantly optimize and improve the healthcare service. It can lead to speeding up the diagnoses and lowering the healthcare costs. Such a system can also be integrated with telepharmacy platforms resulting in increased healthcare efficacy and satisfaction of the patients. Moreover, taking into account the recent situation with the COVID-19 pandemic, the implementation and application of telemedicine into GP's work seem to be an urgent and important task for modern healthcare systems.

### 3.7. 3D Printing for Healthcare and Medical Education

Over the past decade, 3D printing technology has been increasingly used in healthcare. At this point, progress has been made in the field of medical 3D printing, and the technology for manufacturing models of organs and permanent implants has become more reliable [122]. The study conducted by Cheng et al. demonstrated the possibility of improving the behaviour of biodegradable materials used for 3D printing [123]. Although direct printing of tissues and organs is still in its infant stage, domestic and international researchers using printed tissues and organs have begun to study the possibility of 3D printing of organs and blood vessels [124].

In fact, 3D printing technology allows the surgeon to provide a physical 3D model of the desired patient's anatomy site, which can be used to accurately plan access and cross-sectional 3D imaging. In addition, 3D printing makes it possible to select the size of the prosthesis components with high accuracy before implantation. 3D printing can also be used to make custom implants or surgical instruments, thus allowing the individualization of instruments and prostheses without increasing costs [125].

Examples using 3D technology in medicine have been largely discussed in [125] and are briefly listed and commented hereafter: (1) osteoporosis treatment: after pharmacological treatment, 3D printing could be useful to confirm the results; (2) testing of various medical devices; (3) medical education: the advantages of 3D printing are the reproducibility and safety of the 3D printing model (in relation to the dissection of the corpse); the ability to model various physiological and pathological situations from a huge set of data and images, as well as the ability to exchange 3D models between different institutions, especially those that have fewer resources; (4) patient education: patient-oriented care makes patient education one of the top priorities for most health care providers; (5) improving the forensic medical examination: one could use a three-dimensional model to easily demonstrate various anatomical anomalies that jury members may find it difficult to understand using cross-sectional images; (6) 3D printing can be used to produce implantable tissues (synthetic skin printing for transplantation to burn patients); (7) 3D printing has been utilized for testing cosmetic, chemical and pharmaceutical products; (8) another example is replication of heart valves using a combination of cells and biomaterials to control valve stiffness or replication of human ears using forms filled with gel containing bovine cartilage cells suspended in collagen; (9) personalized 3D printing of drugs: 3D printing of drugs consists of printing a layer of powdered medicine so that it dissolves faster than conventional tablets. It also allows to personalize the required amount of medication for the patient; (10) printing of synthetic organs: 3D printing can make it possible to save lives by implanting artificial organs viz artificial hearts in patients who require transplantation and thus reducing the waiting lists. More importantly, printed organs may also be used to replace animal laboratory models for toxicity tests [125].

In addition, 3D printing can be successfully used for medical education as well [126, 127]. It was shown that 3D printed models adjusted for the patient can be used for improving productivity and learning [125, 128]. The 3D printing of anatomical models can reduce the need for cadaveric samples. Moreover, it provides an opportunity to get access to the 3D models of different anatomies from a large set of imaging data. It can also help to exchange 3D models between different institutions, inside and out of the country [129]. Another important advantage of 3D tactile models is simulation training [130]. It was shown that 3D-modeling can be effectively employed for training in various medical specialties such as otorhinolaryngology [131], cardiology [132], general surgery [133], and orthopaedics [134].

## 4. Discussion

The term “digital health” or “e-health” covers a bunch of concepts, approaches, and technologies, such as big data, artificial intelligence, genomics, analytics, telemedicine, smart devices and wearables, and mobile phone health applications [135, 136]. In recent decades, health digital technologies were widely studied and applied in biomedicine, research, and medical education [137]. It encompasses the application of “digital health” concepts for the optimization and the improvement of the diagnosis and treatment of various disorders. Moreover, such technologies became highly sought due to recent pandemics, including COVID-19 [138, 139]. The disastrous dissemination of COVID-19 through all continents triggered the need for fast and effective solutions to localize, manage, and treat the viral infection. In this regard, the use of telemedicine and other e-health technologies might help to decrease the burden from doctors and the national healthcare systems. Such digital platforms can facilitate the diagnosis, making an appointment, and treatment of infected patients, thus, reducing the risk of viral contracting for the physicians and workers of primary healthcare.

Despite the definite advantages of e-health platforms, the hospitals and outpatient clinics predominantly rely on the classic scheme of medical services. The same situation is happening in medical education, where standard methods and approaches remain the main way of teaching medical students and residents. Another factor hampering a wide implementation of digital technologies in healthcare is the lack of funding, particularly from governments. However, the private sector is more eager in the innovations and applications of digital health systems [140]. It is partly due to the flexibility and availability of requested funds in private companies compared to the public healthcare sector. Nevertheless, the situation varies from country to country, even in well-developed western economies. In this regard, the wide and fast implementation of digital health platforms can be more anticipated in developing regions such as Africa and Asia rather than in the Western countries in the near future.

**Figure 4:**
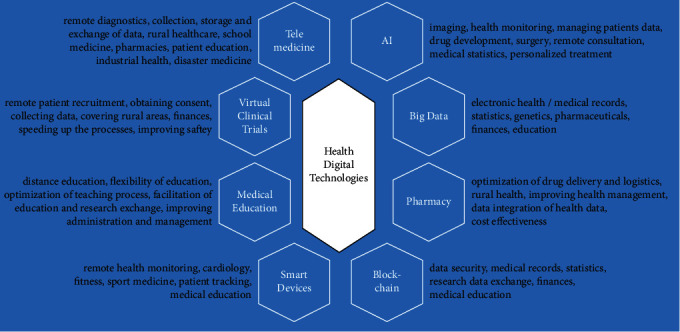
Summary of applications of digital health technologies discussed in the review.

Digital technologies have already had a huge impact on the current healthcare system over the world. In fact, the implementation of different types of digital technologies has led to improving the quality, efficiency, affordability, and accessibility of medical care in different countries. For example, the application of telemedicine has helped to reduce hospital admissions and mortality rates among patients suffering from cardiovascular diseases [141, 142] and diabetes [143, 144]. However, the precise assessment of the effect of digital technologies on healthcare is difficult due to the large scope of proposed technologies presented on the market. Anyway, digital health services have already irreversibly changed the nature and principles of interactions between patients and healthcare workers. Particularly, it has affected the cost-effectiveness, quality, and speed of medical services. In this case, both sides (patient and doctors) win.  Figure 4 provides a summary of applications of digital health technologies discussed in the review.

From a long-term perspective, health professionals will be supported by the power of AI and access to large databases. Such support would improve, optimize, and speed up the diagnosis and treatment. Moreover, the doctors would have an opportunity for continuous medical training and education that is highly crucial for this type of professional activity. In fact, the traditional model of training has been slowly, but steadily, replaced by distance learning that saves time and money. On the other hand, the patients will benefit from improved communication with the physicians and access to the medical information. In addition, the wide implementation of wearables and smart monitoring devices would improve the medical service and develop the basis for preventive and personalized medicine in the near future.

However, despite the promise that such digital solutions can help to decrease the workload of healthcare workers, the potential benefits can be accompanied by negative and unpredictable impacts, especially in long-term perspectives. In this regard, the application, ethical aspects, social, mental, and financial factors of digital health technologies should be carefully analyzed and validated. In fact, there is a range of various challenges, difficulties, or concerns in relation to the development, integration, and implementation of novel technologies and methods in the healthcare systems. It encompasses the reliability and accuracy of medical digital devices, availability, and free access to costly equipment, digital literacy of the patients and doctors along with their ability to efficiently operate the smart devices and digital platforms. Another main concern is related to the fears that digital technologies could replace doctors. It is related to the ability of machines to self-educate and improve their performance, particularly in medical image recognition and analysis. In fact, some medical professions will soon experience the impact of digital health technologies such as in psychiatry, forensic medicine, pathology, neurology, radiology, and pharmacology. It has been dictated by the rapid introduction of AI-based methods and approaches in these disciplines.

The ethics issue is another challenge related to digital health. The situation is complicated due to the participation (in the development of digital platforms) of various types of stakeholders, including big and small technological companies, universities, healthcare providers, patients, and public organizations. Thus, the integrity and effectiveness of health digital platforms mainly depend on the responsibility and moral principles of the participants in the digital health market. Nebeker et al. highlighted five main domains of the digital health decision-making domain framework designed for health research and clinical applications: (1) participants privacy, (2) risks and benefits, (3) access and usability, (4) data management, and (5) ethical principles [145].

Privacy encompasses security, confidentiality, discrimination, unintended uses of medical information, and the right of patients to know how their data will be used [146]. To the present, there are no universal ethical regulations of health data protection [147]. Nevertheless, there is a set of practices and recommendations for health organizations, including the Health Insurance Portability and Accountability Act (HIPAA, USA), Health Information Technology for Economic and Clinical Health Act (HITECH), Office of the Australian Information Commissioner (OIAC, Australia), Personal Information Protection and Electronic Documents Act (PIPEDA), and General Data Protection Regulation (GDPR, EU). Therefore, private companies or governmental health service-providers must take into consideration such recommendations and the ethical issues related to the utilization of digital health data.

The privacy can be optimized and enhanced through establishing trust with patients and facilitating the sharing of data between healthcare stakeholders, using where appropriate deidentification technologies covering the whole lifecycle of data collection, governance, and handling [148]. The digital health systems must be oriented on patients' needs and privacy, foremost if it concerns patient personal information and its utilization.

Access, availability, and costs of health digital services have been the topics of discussion in the media and scientific literature during recent decades [146, 149]. There is fear that digital health will be accessible only for a wealthy cohort of the population [145]. Nonetheless, the recent rise of health mobile apps in developing parts of the world such as Africa gives hope that the digitalization of healthcare will be an integral part of ordinary people everywhere regardless of their income, race, and social status.

Apart from the financial aspect of digital health, there is also a problem of the availability of such services for vulnerable and often neglected parts of the population, including the homeless, elderly, disabled persons, and patients suffering from rare disorders [145, 146]. Brall et al. stressed out that the developers of digital health platforms have a moral responsibility to devise such systems taking into account ethical aspects to satisfy and represent all parts of the population and leave no ground for bias and resulting discrimination [146].

## 5. Challenges

The digital revolution in healthcare is facing many challenges and questions. “It encompasses the need for building tools and services supporting the digital environment and for restructuring the current traditional medical systems and classic approaches.” It is a crucial point not only for undeveloped countries but also for the Western world as well. The second question is related to actual benefits from digital health technologies for healthcare workers and patients as well. In fact, most of the proposed digital health technologies lack an evidence base.

Moreover, some digital health products available on the market are not able to clearly demonstrate clinical relevance and benefits for medical professionals. The question is May digital health technologies optimize and enhance the effectiveness, speed, quality, and affordability of healthcare? Also in limited resources subsets? Another issue is related to the lack of concrete clinical guidelines for the application of digital health in clinical practice and medical education. The last but not the least very important issue is disconnection and lack of close communication between the industry, innovators, investors, and practicing healthcare professionals.

## 6. Conclusions

To summarize, the digital revolution has been affecting and reshaping health care systems worldwide. It encompasses the changes of the fundamental principles and approaches of medical service and education. It was shown that the application of novel digital innovations can improve the accessibility, quality, and flexibility of healthcare for the public not only in Western countries but also in developing countries as well. For example, telemedicine helps people from rural and remote regions to get high-quality consulting, diagnostics, and treatment.

However, there is a range of questions to be answered prior to the wide implementation of digital health platforms. It encompasses the clinical effectiveness of the proposed technologies and their validation. The second issue is the reliability and safety of such digital health innovations. It implies meticulous testing and set-up clinical studies according to ethical principles [145]. Due to the lack of official regulations and recommendations, the stakeholders, including private and governmental organizations, are facing the problem of adequate validation and approbation of novel digital health technologies. In this regard, proper scientific research is required before a digital product is deployed for the healthcare sector [145].

### 6.1. Study Limitations

This review highlights and analyzes the application of big data, artificial intelligence, telemedicine, block-chain platforms, smart devices in healthcare, and medical education. Due to the huge scope of the area on digital health technologies and education, the review was not able to discuss all aspects of these fields. The review is aimed at discussing the recent advances in medical technologies and providing examples of the relevant studies.

## Figures and Tables

**Table 1:** Examples of AI applications in healthcare.

Applications	Study aims	Outcomes	References
Ultrasound imaging	Development of deep learning detection network for ultrasonic equipment for real-time detection of breast cancer.	Method to realize the intelligence of the low-computation-power ultrasonic equipment, and real-time assistance for detection of breast lesions was developed.	[12]..................

CT imaging	To perform a quantitative and qualitative evaluation of a deep learning image reconstruction (DLIR) algorithm in contrast-enhanced oncologic CT of the abdomen.	DLIR improved CT evaluation of the abdomen in the portal venous phase. DLIR strength should be chosen to balance the degree of desired denoising for a clinical task relative to mild blurring.	[13]..................

MRI	To develop a deep learning algorithm for automated detection and localization of intracranial aneurysms on time-of-flight MR angiography and evaluate its diagnostic performance.	A deep learning algorithm detected intracranial aneurysms with a high diagnostic performance which was validated using an external data set.	[14]..................

Cancer diagnosis	To conduct the breast cancer diagnosis by using principal component analysis-support vector machine (PCA-SVM) and principal component analysis-linear discriminant analysis-support vector machine (PCA-LDA-SVM) model classifier algorithms (LabVIEW).	The proposed method provides improvement especially for the polynomial kernel function. An increase in classification accuracy was observed in the test phase compared to PCA-SVM, along with improved classification.	[15]..................

Cancer diagnosis	To develop a computerized image analysis system using deep learning for the detection of esophageal and esophagogastric junctional (E/J) adenocarcinoma.	AI system achieved high sensitivity and acceptable specificity for the detection of E/J cancers and may be a good supporting tool for the screening of E/J cancers.	[16]..................

Cancer diagnosis	To study whether an artificial intelligence (AI) system can increase the accuracy of characterizations of polyps by endoscopists of different skill levels.	The method significantly increased the accuracy of evaluation of diminutive colorectal polyps and reduced the time of diagnosis by endoscopists.	[17]..................

Drug development	To study whether recurrent neural networks can be trained as generative models for molecular structures, similar to statistical language models in natural language processing.	Recurrent neural networks based on the long short-term memory (LSTM) can be applied to learn a statistical chemical language model. The model can generate large sets of novel molecules with physicochemical properties that are similar to the training molecules ones.	[18]..................

Genomics	To validate the ability of a computational approach based on deep neural networks (DeepCpG) to predict methylation states in single cells.	DeepCpG yields substantially more accurate predictions than old methods. It was shown that the model parameters can be interpreted, thereby providing insights into how sequence composition affects methylation variability.	[19]..................

**Table 2:** List of examples of the applications of smart devices for heart monitoring.

Cardiologic applications	Proposed device	Outcomes	References
Electrocardiogram (ECG)	Wearable ECG measurement device (smart clothes)	The best electrode positions to be used to measure ECG signals by means of a two electrodes recording system were identified, and the presented wearable measurement device can obtain good performance when one person is under the conditions of sleeping and jogging.	[33]..................

Electrocardiogram (ECG)	Wearable smartphone-enabled cardiac monitoring device (smart sock)	The home use of smartphone-enabled technology to monitor the neonatal and infant cardiac heart rate can identify asymptomatic arrhythmias.	[34]..................

Electrocardiogram (ECG)	Wearable ECG device.	The study demonstrated the feasibility of real-time AF detection by means of a wearable ECG device. It constitutes a promising step towards the development of novel ECG monitoring systems to tackle the growing AF epidemic.	[35]..................

Electrocardiogram (ECG)	Smart 12-lead ECG acquisition T-shirt.	A smart T-shirt with 13 textiles electrodes allows short-duration 12-lead ECG acquisition with quality levels comparable to Holter recordings. The novel device should now be evaluated for long-term noninvasive ECG monitoring.	[36]..................

Heart rate	Wrist-worn heart rate (HR) monitor.	HR accuracy of two commercially available smart watches [SW] (Fitbit charge heart rate [FB] and apple watch series 3 [AW]) was compared with Holter monitoring in an ambulant patient cohort. The smart watches underestimated heart rate in AF particularly at heart rate ranges > 100 bpm.	[37]..................

Heart rate and blood pressure	Wireless pulse and blood pressure monitoring system.	A wireless blood pressure monitoring system was designed and implemented for a smartphone-based management unit with Graphical User Interface (GUI) and database.	[38]..................

Heart rate	Wrist-worn device.	The study explored the feasibility to estimate heart rate recovery parameters after stair climbing using a wrist-worn device with embedded photoplethysmography and barometric pressure sensors. The proposed approach to monitoring heart rate recovery parameters in an unobtrusive way that may supplement the information provided by personal health monitoring devices.	[39]..................

Electrocardiogram (ECG)	Wearable sensor device (Bioharness 3.0 by Zephyr).	SVM-based algorithm designed to detect atrial fibrillation (AF). The results showed a sensitivity of 78% and a specificity of 66%, making this version of e-health system suitable for real-time monitoring of AF events.	[40]..................

Heartbeat and cardiac-related motions of the chest	Smart textile-based on fiber Bragg grating (FBG) sensor.	The study demonstrated the capability of the proposed smart textile to monitor cardiac activity at different measurement points. The evaluation of the influence of measurement sites on the signal amplitude can be considered as a first effort to drive the standardization of sensor positioning on the chest.	[41]..................

## Data Availability

All relevant data are within the paper.
